# Theoretical design of a space bioprocessing system to produce recombinant proteins

**DOI:** 10.1038/s41526-023-00324-w

**Published:** 2023-09-16

**Authors:** Mathangi Soundararajan, Matthew B. Paddock, Michael Dougherty, Harry W. Jones, John A. Hogan, Frances M. Donovan, Jonathan M. Galazka, A. Mark Settles

**Affiliations:** 1 KBR, NASA Ames Research Center, Moffett Field, Mountain View, CA 94035 USA; 2 Bioengineering Branch, NASA Ames Research Center, Moffett Field, Mountain View, CA 94035 USA; 3 Space Biosciences Division, NASA Ames Research Center, Moffett Field, Mountain View, CA 94035 USA

**Keywords:** Biomaterials - proteins, Aerospace engineering

## Abstract

Space-based biomanufacturing has the potential to improve the sustainability of deep space exploration. To advance biomanufacturing, bioprocessing systems need to be developed for space applications. Here, commercial technologies were assessed to design space bioprocessing systems to supply a liquid amine carbon dioxide scrubber with active carbonic anhydrase produced recombinantly. Design workflows encompassed biomass dewatering of 1 L *Escherichia coli* cultures through to recombinant protein purification. Non-crew time equivalent system mass (ESM) analyses had limited utility for selecting specific technologies. Instead, bioprocessing system designs focused on minimizing complexity and enabling system versatility. Three designs that differed in biomass dewatering and protein purification approaches had nearly equivalent ESM of 357–522 kg eq. Values from the system complexity metric (SCM), technology readiness level (TRL), integration readiness level (IRL), and degree of crew assistance metric identified a simpler, less costly, and easier to operate design for automated biomass dewatering, cell lysis, and protein affinity purification.

## Introduction

Liquid amine scrubbing is a mature post-combustion carbon capture technology on Earth^1–4^ and is a promising approach to scrub CO_2_ produced during crewed space missions^5–8^. The Thermal Amine Scrubber on the International Space Station (ISS) is one of three candidate technologies for CO_2_ removal and has been successfully operated in space^7,8^. In these systems, cabin air containing elevated levels of CO_2_ is passed over an organic liquid amine which absorbs the CO_2_ as carbamate or HCO_3_^−^ depending on the solvent. Pure CO_2_ is recovered by raising the solution temperature and regenerates the amine for subsequent capture at low temperature^9^. An efficient liquid amine system should have fast absorption kinetics to reduce system size as well as a low desorption temperature to minimize energy inputs. Different liquid amines either have fast absorption kinetics or low desorption temperature but not both simultaneously^10^. Carbonic anhydrase is an enzyme that catalyzes the interconversion of CO_2_ and HCO_3_^−^ to increase the CO_2_ sequestration in some liquid amine systems. Addition of carbonic anhydrase enhances the absorption kinetics of liquid amines with a low desorption temperature enabling both reduced size and improved energy efficiency of CO_2_ scrubbing^11–13^. Thermostable enzymes with high pH tolerance have been identified and developed to improve the activity of the enzyme in the harsh conditions of a liquid amine system^11,12^.

Implementing an enzyme-assisted liquid amine CO_2_ scrubber requires a time-course supply of carbonic anhydrase due to loss of enzyme activity from the thermal cycles. On long-duration space missions close to Earth, this requirement could be met by resupply or long-term storage. However, resupply is not an economical option for many deep space missions like those planned for Mars. Purified enzymes are typically sensitive to room temperature conditions and require ultra-low storage temperatures to retain activity long-term. Not all enzymes retain activity in low temperature storage and in situ production of enzymes would mitigate risks of relying solely on low temperature storage of proteins with limited stability. Space biomanufacturing systems have the potential to produce enzymes and other biological materials using in situ resources during a Mars mission^14–19^. Space systems must minimize cost and crew time, while assuring astronaut safety and addressing effects of increased radiation and reduced gravity^20–23^.

Previous space biomanufacturing studies and reviews evaluated large-scale mission design^14,24,25^, microbial growth kinetics^26^, and bioreactor design^26–28^. Extracting products of interest at sufficient quality is equally essential to develop biomanufacturing. Systems such as Wetlab-2 or the Gene Expression Measurement Module (GEMM) illustrate the challenge of adapting biological sample processing for RNA extraction and molecular analysis in the microgravity environment^29,30^. In these systems, the sample mass and volume processed was small with the goal of providing biological inputs for analytical experiments^29,30^. Future space biomanufacturing systems need to address post-growth processing at larger scales to extract products for in situ use.

In this study, we compared commercial technologies and potential designs for in space biomanufacturing systems. Our operational scenario was post-growth bioprocessing to produce recombinant carbonic anhydrase from *Escherichia coli* during a Mars mission. Since carbonic anhydrase is unlikely to be the only useful product in deep space missions, the ability to produce a variety of recombinant proteins from multiple chassis organisms was a key consideration for the designs. The designs were qualitatively compared using non-crew time equivalent system mass (ESM) analysis^20,31^, a system complexity metric (SCM)^22^, technology readiness level (TRL)^32^, integration readiness level (IRL)^33,34^, and number of crew assisted steps (degree of crew assistance) to guide future prototype development efforts.

## Results

Bioprocessing technology comparisons were based on a production scenario that required thermostable and high-pH tolerant carbonic anhydrase for a liquid amine CO_2_ capture system during 600 days of surface operations on Mars^35,36^. In this system, enzyme activity will decay with heating-cooling cycles, and will require intermittent addition of recombinant protein to supply sufficient enzyme activity throughout the surface operations. Protein purification was assumed to be required to reduce side reactions with the liquid amine; however, multiple chromatography steps to produce highly purified protein are not required for this application^11^. The specific enzyme considered is an engineered version of the *Desulfovibrio vulgaris* carbonic anhydrase with a 6x-His tag expressed using *E. coli*^11,12^. The enzyme variant was selected for activity in N-methyldiethanolamine and long-term stability at 50 °C. Intracellular production of this thermostable enzyme has been demonstrated *E. coli*, while extracellular production has not been reported. A total of ~14 g of active enzyme was estimated to be required to supply a crew of six for 600 days. The calculation assumed a 10 L liquid amine reactor requiring 0.2 g L^−1^ of active enzyme^11^. Based on the reported 6–14 week half-life of the thermostable variant, weekly supplementation of 0.1 to 0.2 g L^−1^ of fresh active enzyme would be required^11^. A prior study reported 180 mg L^−1^ of recombinant protein yield using acetate as the carbon source^37^. Assuming a 20% loss during post-growth processing, 100 cultures at 1 L volume is estimated to be sufficient.

### Potential workflows for the operational scenario

**Fig. 1:**
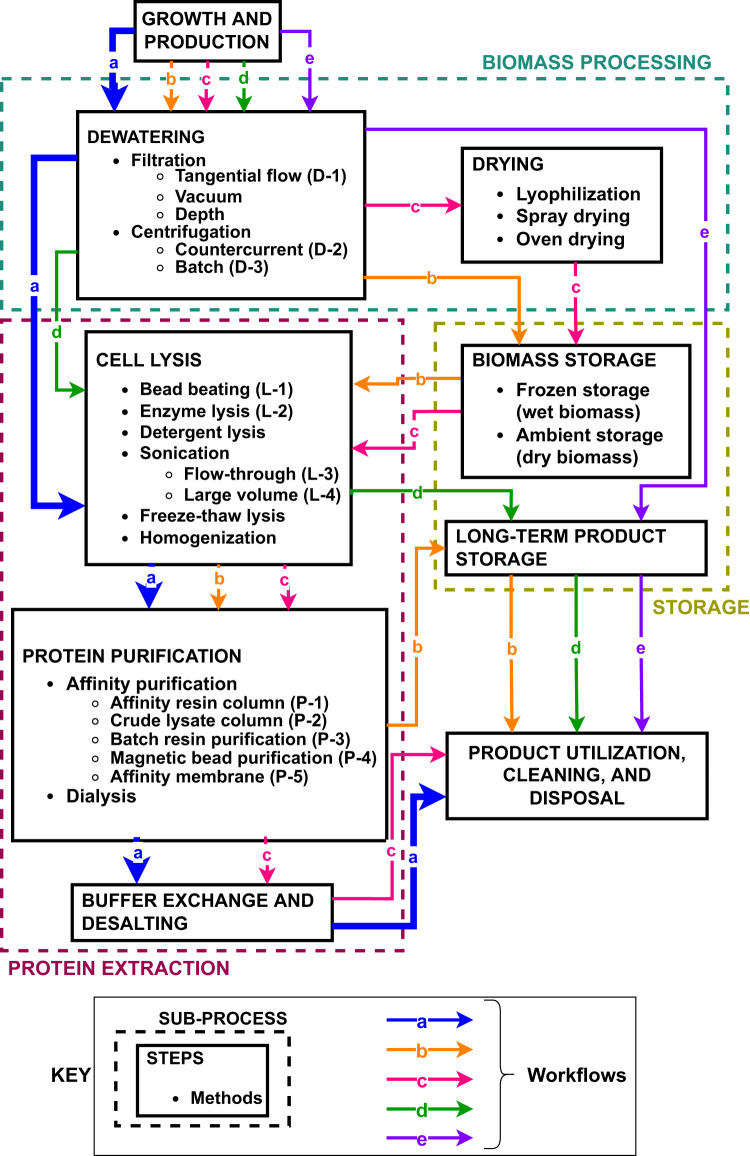
Flow diagram of potential bioprocessing strategies. Dashed boxes group the primary sub-processes of biomass processing, protein extraction, and product storage. Solid boxes give individual steps with bulleted lists of common methods to complete the step. Methods compared in this analysis have parenthetical abbreviations that are used in Figs. 2, 3. Lettered arrows (a, b, c, d, e) give examples of possible workflows for processing *E. coli* cells expressing recombinant carbonic anhydrase.

Figure 1 shows five potential bioprocessing workflows (a, b, c, d, and e) starting from a common cell growth and production step. Each workflow considers sub-processes of biomass processing, protein extraction, and storage. These sub-processes were split into steps that could have multiple alternate methods. For the sub-process of biomass processing, dewatering and drying steps were the primary options considered. The stated use case will produce recombinant protein intracellularly and will require a protein extraction sub-process including cell lysis, protein purification, and buffer exchange/desalting steps. Finally, the protein product could have a storage sub-process, either as biomass or as a purified product.

Workflow-a was selected as best aligned with the operational scenario of supplying purified carbonic anhydrase on a 6 to 8-day cycle. This workflow moves from growth and production steps to dewatering, cell lysis, protein purification, and buffer exchange to end with product utilization, cleaning, and disposal. Inclusion of a modular lysis step allows for the processing of both intracellular and extracellular products. Although storage and drying steps were considered, they were deemed unnecessary based on the selected scenario. There are no steps in Workflow-a that are unique requirements of the space production scenario, and a bioprocessing system design that enables this workflow is predicted to be useful for recombinant protein production in terrestrial applications.

### Biomass processing

**Fig. 2:**
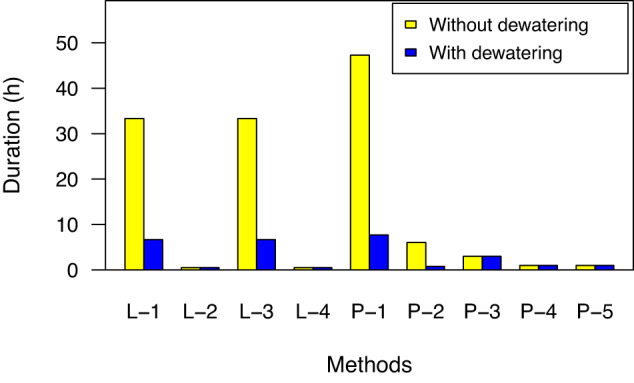
Estimated process duration for each run of the lysis (L) and purification (P) based on recommended manufacturer protocols or published literature. Methods included: flow through bead beater (L-1), enzyme lysis (L-2), flow cell sonicator (L-3), large volume probe sonicator (L-4), affinity resin column (P-1), crude lysate column (P-2), batch affinity resin (P-3), batch affinity magnetic beads (P-4), and affinity membrane (P-5). Yellow bars are process times of 1 L culture and blue bars are process times of 200 mL concentrated biomass. See supplementary data 1 for detailed calculations.

Dewatering cultures greatly reduces processing volumes for protein extraction or biomass storage sub-processes. However, it may be feasible to complete protein extraction without a dewatering step. We modeled the impact of dewatering on cell lysis and protein purification steps by scaling processing time for flow-through methods including bead beater lysis (L-1), flow cell sonication (L-3), affinity resin column purification (P-1), and crude lysate column purification (P-2) methods (Fig. 2, Supplementary data 1). This analysis showed that processing cultures without dewatering would require multiple days using flow-through methods.

Other methods could be completed in batch including enzyme lysis (L-2), large-volume probe sonication (L-4), batch affinity resin purification (P-3), magnetic bead affinity purification (P-4), and affinity membrane purification (P-5). These were scaled by increasing materials to limit impacts on processing duration (Fig. 2). However, most protein purification methods were developed for cell lysates from concentrated biomass suspensions. It is unknown whether cultures could be lysed directly, or if large-scale batch purification would yield sufficiently concentrated purified protein. We concluded that dewatering would help ensure feasibility for batch methods and allow flow-through methods to be considered as options in the bioprocessing designs.

**Fig. 3:**
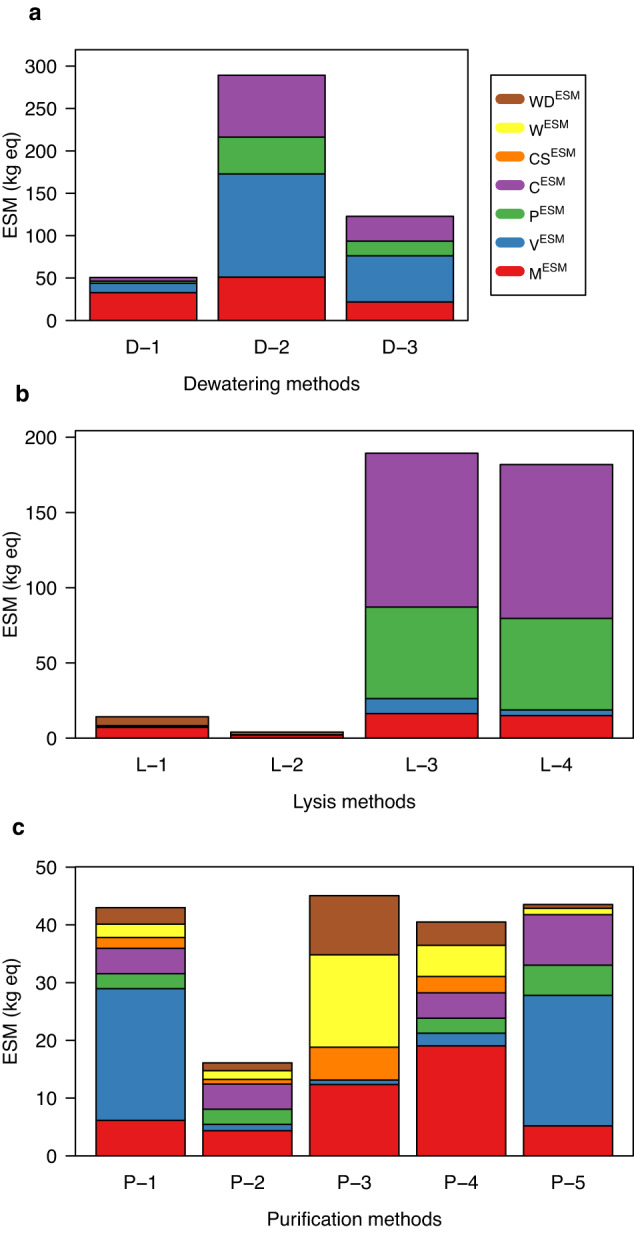
Non-crew time ESM models assuming 100 bioprocessing runs. **a** Dewatering methods included tangential flow filtration (D-1), counterflow centrifugation (D-2), and batch centrifugation (D-3). **b** Cell lysis methods included bead beating (L-1), enzymatic lysis (L-2), flow through sonication (L-3), and large volume probe sonication (L-4). **c** Protein purification methods included clarified lysate column (P-1), crude lysate column (P-2), batch resin purification (P-3), magnetic bead purification (P-4), and affinity membrane purification (P-5). Each bar graph shows mass equivalencies for: total mass (M^ESM^), volume (V^ESM^), power (P^ESM^), cooling (C^ESM^), cold storage (CS^ESM^), water treatment (W^ESM^), and solid waste disposal (WD^ESM^). See Table 2 for ESM values and Supplementary data 1 for detailed calculations.

Non-crew time ESM is a metric to estimate the flown mass required to implement a space technology and can help guide selection of alternative technologies^20,31,38^. The metric is a linear model to estimate mass of the system along with mass equivalences for providing volume and other infrastructure within a spacecraft (Supplementary data 1). Table 1 gives the 2015 and 2022 equivalency factors used to calculate ESM^39,40^, and Table 2 reports the non-crew time ESM for the methods and bioprocessing designs. These calculations were highly correlated (*r* = 0.992), and values using the 2015 equivalency factors are reported in the figures to enable comparisons to prior technology proposals. Due to the variability in calculating ESM for preliminary designs, the metric is only used for selection of competing technologies when ESM estimates approach a 5- to 10-fold difference^31,41^. Figure 3a shows ESM calculations for dewatering methods. Batch and counterflow centrifugation have ~5-fold higher ESM than a tangential flow filter. Despite the similar ESM of batch and counterflow centrifuges, a counterflow centrifuge is easier to automate and can potentially reduce crew time requirements. Based on these considerations, we removed batch centrifugation from further consideration.

**Table 1:** Mars surface equivalency factors for this study.

Parameter	2015 Equivalency factor	2022 Equivalency factor
Shielded Volume (V)	215.5 kg_eq_ m^−3^	79.3 kg_eq_ m^−3^
Power (P)	87 kg_eq_ kW^−1^	162 kg_eq_ kW^−1^
Thermal Control (C)	146 kg_eq_ kW^−1^	96 kg_eq_ kW^−1^
Cold Storage (CS)^1^	0.79 kg_eq_ kW^−1^	0.21 kg_eq_ kg^−1^
Water Treatment (W)^1^	0.12 kg_eq_ kg^−1^	0.3 kg_eq_ kg^−1^
Waste Storage (WS)^1^	0.83 kg_eq_ kg^−1^	0.93 kg_eq_ kg^−1^

^1^Infrastructure externality factor; see Methods and Supplementary data 1 for detailed assumptions and calculations.

**Table 2:** Total ESM for the methods and designs reported in this study.

Method/design	2015 factors	2022 factors	Abbreviation in figures
Tangential flow filter	50	40	D-1
Counterflow centrifuge	290	220	D-2
Batch centrifuge	120	90	D-3
Bead beating	14.2	14.4	L-1
Enzyme lysis	4.1	2.6	L-2
Sonicator (flow cell)	189	201	L-3
Sonicator (large volume probe)	182	197	L-4
Affinity resin column	43	32	P-1
Crude lysate column	16	18	P-2
Batch resin purification	46	66	P-3
Magnetic beads	41	46	P-4
Affinity membrane	44	32	P-5
Design 1	460	400	Design 1
Design 2	360	340	Design 2
Design 3	520	450	Design 3

### Protein extraction

ESM models for lysis eliminated sonication due to high power and cooling requirements (Fig. 3b). Although enzyme lysis has a low ESM in this analysis, there are several concerns for implementation of an enzyme-only method that could not be easily modeled via ESM. Lysozyme is only effective as a lysis method for a limited number of microbial host species. The enzyme is most effective when combined with chelators, detergent, or sonication to disrupt the outer membrane of gram-negative bacteria like *E. coli*^42^. The chelators and detergents required for more effective enzyme lysis also create wastewater treatment challenges. Finally, enzyme lysis is not as effective as mechanical lysis^43^ and would likely require greater biomass growth to achieve equivalent recombinant protein yield.

Mechanical lysis with bead beating is a well-established cell disruption technique for a large variety of organisms and developmental stages including spores^44^. A small footprint, flow-through bead beater has been used on the ISS for biology research, demonstrating technology feasiblity^29^. Based on these factors, flow-through bead beating was selected as the lysis method for design comparisons.

ESM estimates for the protein purification step compared five commercial affinity purification methods. ESM estimates were within a 3-fold range for all technologies (Fig. 3c). Like the cell lysis step, we considered feasibility of implementation to select three methods for bioprocessing system design comparisons. The affinity resin column and crude lysate column use equivalent flow-through approaches, but the affinity resin column required a clarified lysate with minimal cell debris. The crude lysate column was selected for increased reliability and reduced complexity. Purification using magnetic beads requires a crew-assisted step. By contrast, the batch resin method would be simpler to automate and was selected. The affinity membrane was selected to analyze design requirements for an alternate solid matrix.

### Bioprocessing system integrated designs

**Fig. 4:**
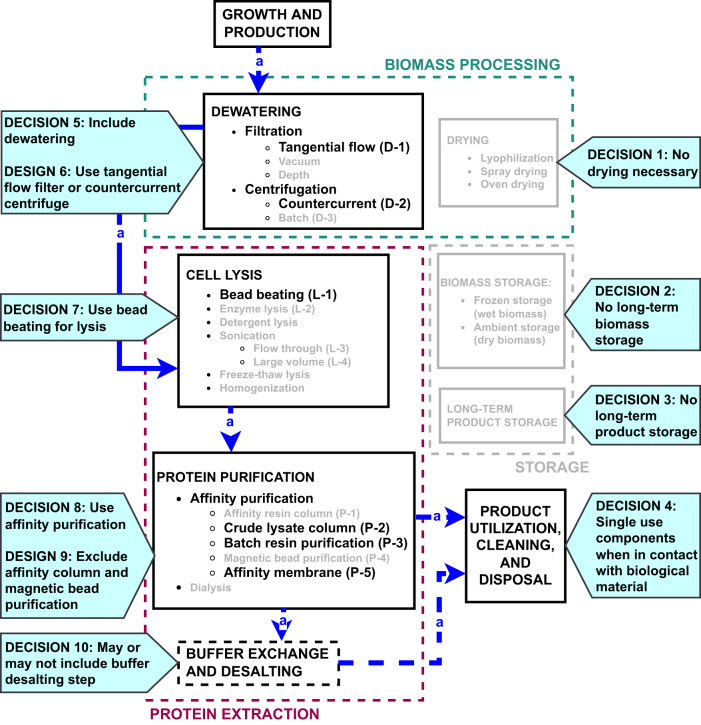
Flow diagram of bioprocessing strategies showing selected methods for integrated, in space designs. Decisions from the trade study are indicated with blue callouts. The rationale for each decision is given in Table 3.

Figure 4 shows the steps and methods eliminated and retained to design integrated bioprocessing systems. Table 3 summarizes key decisions with the resource- and risk-reduction rationale for selection of specific steps and methods. Complex systems with unneeded steps were assessed to have greater risk of failure and was the basis for most of the design decisions. Biomass storage, product storage, and drying steps were eliminated. Cold storage of the biomass and CA product would be desirable to reduce risk for an enzyme-assisted liquid amine scrubber but developing dedicated storage steps in the bioprocessing system added unnecessary complexity. For dewatering, the counter current centrifuge and filtration methods were retained to reduce crew assisted steps. For cell lysis, only a flow-through bead beater was retained to ensure multiple microbial chassis organisms could be processed. For protein purification, crude lysate column, batch resin purification, and affinity membrane purification were retained as methods with lower complexity for implementation.

**Table 3:** Summary of workflow method decisions and rationales.

Decision	Rationale	Issues/concerns
No long-term biomass storage	Reduce complexity	Biomass storage may require cold storage and/or low humidity environment
No long-term product storage	Reduce complexity	Increases mission risk in case of anomalies, but cold storage is unlikely to be available as part of the habitat infrastructure
No drying	Reduce complexity	Possible loss of enzyme activity due to drying
Any component in contact with biological material will be single use	Reduce crew time for cleaning, complexity, and chemical safety concerns	No established cleaning methods for space application
Include dewatering	Shorten processing time	Additional steps add complexity to the system
Use tangential flow filters or counterflow centrifuge	Batch centrifuge requires crew assisted operations	Counterflow centrifuge may require modification for microbial applications
Use bead beating for lysis	Applicable for multiple chassis organisms and demonstrated in space	Sonication requires excessive power; enzyme lysis is inefficient without additional physical cell disruption; bead beater may require modification for large volume processing
Use affinity purification	Product specific, but high efficiency method	Elution requires affinity binding competitors
Exclude affinity column and magnetic beads	Reduce complexity and increase reliability	Affinity column requires lysate clarification, and magnetic beads require magnets for purification steps
May or may not include a buffer exchange and desalting step	Reduce complexity	Additional testing needed to determine if the step is required

**Fig. 5:**
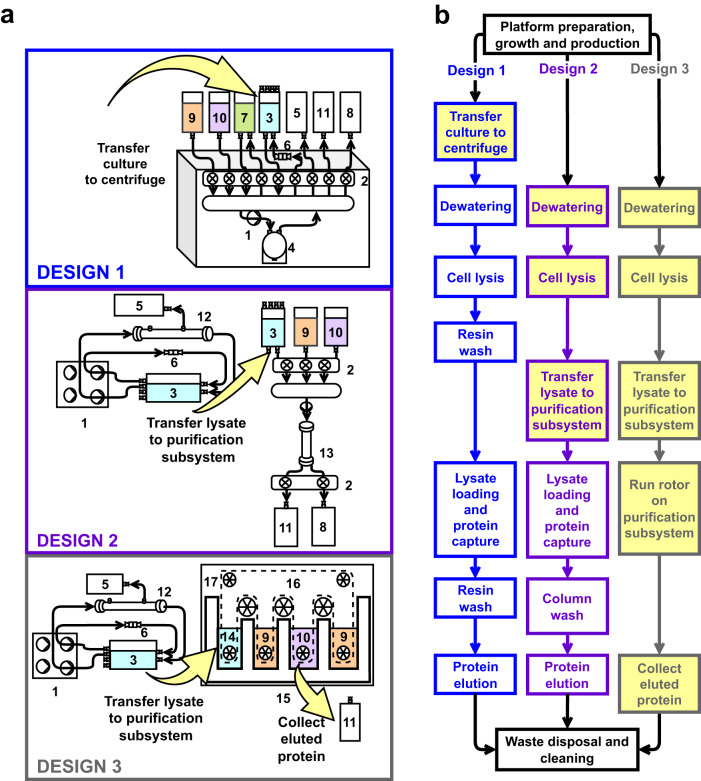
Bioprocessing system designs integrating selected methods for dewatering, lysis, and purification. **a** Design schematics with the following components: peristaltic pump (1), pinch valve (2), biomass reservoir (3), centrifuge cartridge (4), spent media reservoir (5), disposable bead beater (6), affinity resin reservoir (7), waste reservoir (8), wash buffer (9), elution buffer (10), product reservoir (11), tangential flow filter (12), crude lysate column (13), crude lysate chamber (14), affinity purification cartridge (15), affinity membrane (16), rollers (17). Yellow arrows indicate crew-assisted steps. **b** Flow diagram comparing the bioprocessing designs. Yellow boxes indicate operations that require crew-assistance to initiate or complete the operation.

We developed three integrated designs using the methods selected from the trade study (Fig. 5). Design 1 uses a counterflow centrifugation system to dewater biomass, lyse cells, and purify the protein with a batch resin method. Material flow is mediated by a peristaltic pump (1) and automated pinch valves (2). Cells from the biomass reservoir (3) are fed into the centrifuge (4) and concentrated. Supernatant media is collected in a spent media reservoir (5). Concentrated biomass is pumped through a bead beater (6) for lysis and the lysate is returned to the biomass reservoir. The lysate is then pumped into the affinity resin reservoir (7) for protein binding. Protein-bound resin is separated from the lysate using the centrifuge, while the spent lysate is collected in a waste reservoir (8). The resin is washed with buffer (9). Wash buffer is separated by the centrifuge and collected into the waste reservoir. The bound protein is eluted from the resin using elution buffer (10), separated by the centrifuge, and collected in the product reservoir (11).

Design 2 assumes a peristaltic pump (1) from the growth and production step will pump fluids through a tangential flow filter (12) to dewater and concentrate biomass. Concentrated biomass is returned to the biomass reservoir (3), and clarified media is collected in the spent media reservoir (5). The concentrated biomass is lysed by the bead beater (6), and crude lysate is returned to the biomass reservoir. The lysate is transferred to the purification system by the crew. The purification system uses a separate pump (1) and pinch-valve module (2) to load lysate into a crude lysate column (13). The column is washed with buffer (9), and recombinant protein is eluted into the product reservoir (11).

Design 3 uses an identical dewatering and lysis system as in design 2, but with a different protein purification system. Crew will transfer the lysate to the lysate chamber (14) of a continuous loop affinity membrane purification system (15). Rollers (17) move the affinity membrane through buffer chambers for protein binding (14), wash (9), and elution (10). A second wash chamber equilibrates the membrane for multiple cycles of protein binding. The crew transfers the eluted protein to a product reservoir (11).

### Comparative analysis of designs

**Fig. 6:**
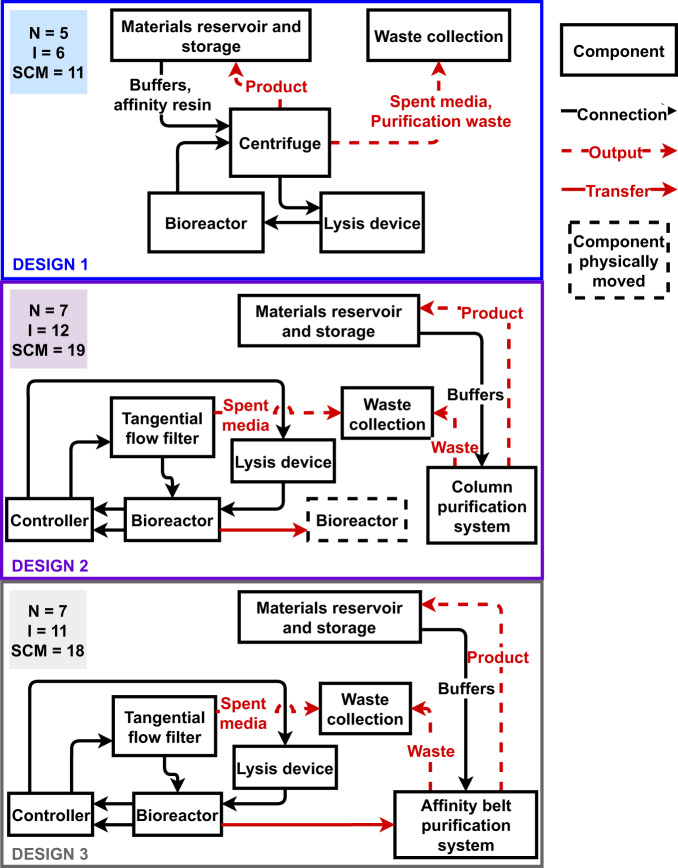
Top-level block diagram of the three bioprocessing designs used to calculate SCM. Major components (N) are in boxes. Individual interconnections (I) are diagrammed with arrows indicating the direction of material flow. Dashed arrows indicate outputs of the bioprocessing systems, and red arrows require crew assistance to complete the actions for the interconnection.

Each design integrates different methods to complete the same bioprocessing steps (Fig. 5b). Multiple systems parameters including ESM, SCM, TRL, IRL, and degree of crew assistance were used to assess the three designs. SCM estimates complexity of life support systems by summing all the components and proposed interconnections of a specific design^22^. Larger SCM values are interpreted as more complex systems with potentially lower reliability. Figure 6 depicts the major components and interconnections used to calculate SCM values for each of the three designs. We also analyzed the degree of crew assistance because crew time was not incorporated into the ESM calculations. Crew-assisted steps are highlighted in yellow or red in Figs. 5, 6.

TRL and IRL use a 9-point scale to assess technology maturity or integration readiness with TRL 9 being spaceflight-proven technologies^32^ and IRL 9 being mission-proven integration^33,34^. Each of the three designs have subsystems for dewatering, lysis, and protein purification steps. The centrifuge for the dewatering step in design 1 was assigned a TRL 4 due to the technology being a commercial product that is demonstrated to automate liquid handling and separate eukaryotic cells in a laboratory or commercial environment. The dewatering subsystems in designs 2 and 3 are identical and assigned a TRL 3. The liquid handling and filtering technologies for this dewatering subsystem have been demonstrated as independent technologies in a laboratory environment making the design proof-of-concept.

The lysis subsystem is identical in all the designs and was assigned a TRL 9 since the bead beater has been demonstrated on the ISS as part of the WetLab-2 hardware^29^. The protein purification steps in designs 1 and 2 use established laboratory affinity purification methods but the designs for automated liquid handling are still at a proof-of-concept. Consequently, these subsystems were assigned a TRL 3. The continuous loop affinity membrane purification subsystem in design 3 is an unproven, innovative concept and was assigned a TRL 2.

The three designs each have two subsystem interfaces, dewatering-lysis and lysis-protein purification. The dewatering-lysis interface of Design 1 was evaluated at IRL 6, established but not validated, since the centrifuge to bead beater interface can be controlled with commercial software but has not been tested. The lysis-protein purification return interface for design 1 is validated with manufacturer protocols and assigned an IRL 7. The dewatering-lysis interface of designs 2 and 3 is identical with a pump connecting the bioreactor bag to the bead beater and were assessed to be at IRL 4 since integration has been checked but automatic control is not yet established. Designs 2 and 3 use manual transfer of the lysate to the protein purification subsystems but the plan for controlling the transfer is not yet established resulting in an IRL 4 for the manual interface.

**Fig. 7:**
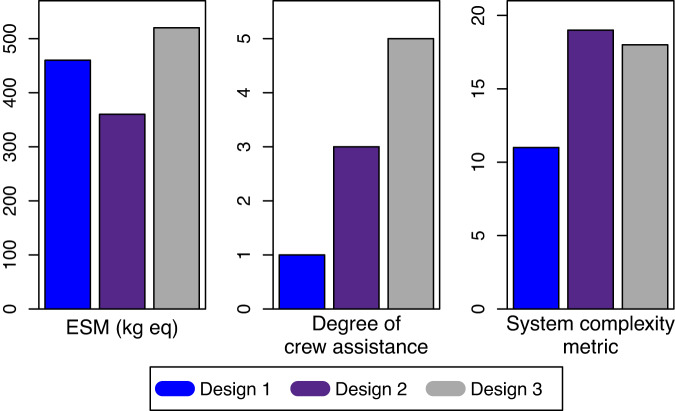
Design metrics for the three integrated bioprocessing designs. See Table 2 for non-crew time ESM values and Supplementary data 2 for detailed ESM calculations.

Figure 7 compares ESM, SCM, and degree of crew assistance for the integrated designs, while Table 4 reports TRL and IRL for the dewatering, cell lysis, and protein purification steps (Supplementary data 2). ESM was comparable for all three designs. Design 1 had the lowest SCM due to the multifunctional commercial counterflow centrifuge that integrates dewatering, cell lysis, and protein purification. The ease of integration and automation in design 1 is also reflected in the low degree of crew assistance and higher average readiness level compared to the other designs. Although these metrics suggest design 1 will be more practical to implement, the differences in the metrics between the designs were not large enough to eliminate specific methods without empirical results.

**Table 4:** TRL and IRL for each bioprocessing step of the integrated designs.

Step	Design 1	Design 2	Design 3
Dewatering TRL	4	3	3
Dewatering-Lysis IRL	6	4	4
Lysis TRL	9	9	9
Lysis-Protein purification IRL	7	4	4
Protein purification TRL	3	3	2
Average readiness level	5.8	4.6	4.4

## Discussion

Carbonic anhydrase improves the efficiency of liquid amine CO_2_ capture systems^11–13^, which is a candidate technology for deep space missions^5–7^. Implementation of an enzyme-assisted liquid amine system requires a steady supply of carbonic anhydrase that may be met by long term storage, resupply, or in situ bioproduction. Long term storage of active enzyme requires low temperature storage conditions, and frequent resupply is not possible for surface missions to Mars. Although carbonic anhydrase production was the use case scenario to guide the system designs, the three design options use research-grade technologies and would be applicable to purify any soluble recombinant protein with an affinity tag from single-cell microbial chassis organisms. The biomass-dewatering and cell-lysis technologies are applicable for both prokaryotic and eukaryotic cells. In the case of a recombinant protein that is secreted into the media, the liquid flow from the biomass dewatering step could be programmed to omit the cell lysis step and route clarified spent media into the affinity purification step.

Bioprocessing system designs for space applications need to optimize mass, efficiency, power consumption, complexity, reliability, and ease of operation^45,46^. The initial space design process largely focuses on ESM to ascertain feasibility either in comparison to existing state of the art or other technologies^18,20,31,47–52^. In this study, we employed numerical metrics like non-crew time ESM along with processing duration, SCM, TRL, IRL, and degree of crew assistance metrics to estimate reliability, potential for implementation, and ease of operation. To maximize the feasibility of the integrated designs, we focused on commercial technologies and materials manufactured for the intended purpose of each step in the bioprocessing workflow.

The numerical metrics used to compare technologies and designs should be considered qualitative indicators. The equivalency factors used to calculate ESM depend upon numerous assumptions for the Mars mission scenario, and a disruptive space technology affecting the relative weighting of mass, volume, power, and cooling would be expected to alter the rankings of the bioprocessing designs. Consequently, we made design decisions based on ESM only when technologies had greater than 5-fold difference in ESM values.

Although SCM, TRL, IRL, and degree of crew assistance are all monotonic functions, these scales are neither linear nor easily transformed into normal distributions for statistical tests. For example, many systems projects successfully reach TRL 4 or 5 but are unable to develop a TRL 6 prototype that can operate in the expected environment. By contrast, systems at TRL 7 more easily continue development through TRL 8 and 9. Thus, the relative difference between starting at TRL 3 versus TRL 4 for advancement to TRL 5 would not be as large as the relative difference between starting at TRL 5 versus TRL 6 for advancement to TRL 7. Likewise, the SCM, IRL, and degree of crew assistance metrics have equal weighting for each additional connection, interface, or step but a subset may be more difficult to engineer or implement. Consequently, the significance of small differences in these metrics needs to be evaluated qualitatively.

Comparisons using non-crew time ESM were able to eliminate batch centrifugation and sonication as methods for integrated designs. However, the integrated bioprocessing designs have a narrow range of 357–522 kg eq ESM. Given these differences are not large enough for system selection, this metric cannot be used to prioritize designs for implementation. The ESM estimates are comparable to a proposed monoclonal antibody production system at 300–1700 kg eq^53^ as well as a plant biomass system for food production at 300–1300 kg eq per kg^16^. For theoretical designs such as the proposed biomanufacturing systems, a difference of less than 10-fold suggests similar infrastructure costs. On a mission-wide basis, the recombinant protein bioprocessing system may be reasonable for a Mars mission. The bioprocessing system adds only 2–3% to the physio-chemical-biological life support system proposed by ESA for a full Mars mission (~18,000 kg eq)^54^.

Crew time and system complexity are important factors in space system design^22,55^. Design 1 ranked best for degree of crew assistance and SCM, which primarily reflects the level of integration for automated fluid handling in the commercial counterflow centrifuge. Additional engineering to integrate pumps and controllers would reduce both SCM and crew-assisted steps for designs 2 and 3. Design 1 also has a higher average readiness level indicating this design may be easier to implement for space, but a limitation of the study is a lack of empirical validation. All components within a design were assumed to be compatible for integration and operate as intended for the specific use case of recombinant protein extraction and purification from *E. coli*. However, there are clear risks for integration and function of each design. The counterflow centrifuge in design 1 needs to be tested for biomass dewatering of microbial cultures. The bead beater proposed in all designs is intended for smaller quantities of biomass and may require modification for the 1 L scale application. The affinity resins proposed in designs 1 and 2 may not have sufficient shelf-life in the manufacturer’s storage conditions, while the conveyor belt affinity membrane in design 3 is still conceptual. Empirical tests are needed to determine the relative efficiency and ease of integration for the selected technologies. We are currently conducting laboratory testing on Earth to serve as a close analog of the intended mission environment with Mars surface gravity. The design decision to use peristaltic pumps for liquid handling decreases the risk of unexpected performance in reduced gravity environments.

Although closed-loop systems and reusability are attractive long-term goals in space, they require increased crew time and system reliability^46,56,57^. On Earth, biomanufacturing often employs single-use technology for various reasons including improved safety as well as reduced contamination, footprint, and cost by eliminating cleaning and sterilization of components that contact biological material^58,59^. Single-use materials for bioprocessing is predicted to reduce crew time and to increase reliability. Consequently, the proposed bioprocessing designs use disposable components in the ESM calculations.

We also identified several factors that could influence a recombinant protein bioprocessing design that were difficult to quantify. For example, there are safety and shelf-life concerns for commercial affinity purification products. Affinity resins are stored in ethanol to prevent biological contamination. Ethanol interferes with the current Environmental Control and Life Support Systems on the ISS, creating a safety risk when large amounts of ethanol are in the cabin air or wastewater. The ethanol risk could be mitigated with an affinity membrane that is stored dry. Both commercial resins and affinity membranes have 1–2 year shelf lives, while a Mars mission is expected to require a 5-year shelf life for all systems^35^. Removing incompatible chemicals and extending the shelf life of the affinity matrix are both critical to advance space biomanufacturing of recombinant proteins.

It is possible that using different strategies to express recombinant proteins could reduce complexity and system mass. For instance, an affinity resin “teabag” can purify proteins secreted into the media without dewatering or lysis steps^60^. Although this strategy would simplify bioprocessing, it limits production to recombinant proteins that have activity after secretion. Moreover, *E. coli* secreted proteins are targeted to the periplasmic space and require an osmotic shock step to release protein into the media. Adding an osmotic shock still requires dewatering to concentrate biomass and the osmotic shock step would replace the cell lysis step. Yeast production hosts such as *Komagataella phaffii* or *Saccharomyces cerevisiae* can secrete recombinant proteins directly into media. These yeast species have larger cell size than *E. coli*, which reduces risk for inefficient or clogged dewatering, but the eukaryotic secretory pathway also post-translationally modifies proteins. Production of active, thermostable CA enzyme would need to be demonstrated to consider these alternate host species.

This study illustrates multiple approaches to rank technologies for design of integrated systems intended for space applications. The extreme environment of space, high cost of launching materials, and limited crew time drive systems to be automated and to minimize mass, power, and volume. The NASA Space Synthetic Biology project is funded to develop a prototype bioprocessing system based on Workflow-a. This on-going work is expected to identify technologies that can be integrated and automated reliably, which could be translated to improved efficiency for industrial biomanufacturing on Earth.

## Methods

The operational scenario investigated was a six person crew on a 3-year Mars mission with 600 days of surface operations^39^. A total of ~14 g of recombinant carbonic anhydrase was estimated to be required to maintain a liquid amine CO_2_ scrubber with active enzyme throughout surface operations. Affinity purification of the enzyme was assumed to be needed to reduce liquid amine side reactions with cellular debris. Sufficient carbonic anhydrase could be supplied by approximately 80 bioprocessing runs using 1 L cultures with a yield of 180 mg purified enzyme per run. Supplies and power for a total of 100 production runs were assumed to give sufficient redundancy for production runs that failed to meet expected yield.

A review of recombinant protein purification from unicellular microorganisms identified sub-process steps (Fig. 1). Essential steps for the operational scenario, such as lysis or purification, were retained. Biomass dewatering was investigated as an optional step. Non-essential steps, such as storage and drying, were eliminated to simplify potential workflows. A trade study of potential methods for each sub-process step was conducted, and non-crew time ESM was used to evaluate individual technologies for in-space application. Technologies with comparable ESM were used to develop three designs, which were analyzed using four metrics described below.

### Non-crew time ESM metric

Non-crew time ESM was calculated using Eq. 1 based on guidelines from Levri, et al.^20^.

1 $$\begin{array}{ll}{\rm{ESM}}=\sum \left[{M}_{{\rm{i}}}+{V}_{{\rm{i}}}\cdot {V}_{{\rm{eq}}}+P\cdot {P}_{{\rm{eq}}}+C\cdot {C}_{{\rm{eq}}}+D\left({M}_{{\rm{t}}}+{V}_{{\rm{t}}}\cdot {V}_{{\rm{eq}}}\right.\right. \\ \left.\left.\qquad\qquad+\,{\rm{CS}}\cdot {{\rm{CS}}}_{{\rm{eq}}}+W\cdot {W}_{{\rm{eq}}}\cdot +{\rm{WS}}\cdot {{\rm{WS}}}_{{\rm{eq}}}\right)\right]\end{array}$$

Where, *M*_i_ is initial mass (kg), *V*_i_ is initial volume (m^3^), *V*_eq_ is volume equivalency (kg eq m^−3^), *P* is power consumed (kW), *P*_eq_ is power equivalency (kg eq kW^−1^), *C* is thermal cooling (kW), *C*_eq_ is thermal cooling equivalency (kg eq kW^−1^), *D* is mission duration (number of runs), *M*_t_ is time dependent mass (kg), *V*_t_ is time dependent volume (m^3^), CS is cold storage mass (kg), CS_eq_ is cold storage equivalency (kg eq kg^−1^), *W* is water mass required per run (kg), *W*_eq_ is water treatment equivalency (kg eq kg^−1^), WS is solid waste mass per run (kg), and WS_eq_ is solid waste storage equivalency (kg eq kg^−1^).

Crew time was excluded from all ESM calculations due to the large uncertainty in estimating crew time requirements without empirical tests of the integrated designs. Equation 1 includes core non-crew time ESM components for mass, volume, power, and cooling as well as estimates of working mass for the cold storage, water, and waste storage systems that were determined to be externalities of all the bioprocessing designs. Parameters for volume (V^ESM^), power (P^ESM^), and thermal cooling (C^ESM^) used equivalency factors estimated for nominal crewed surface missions to Mars from both 2015 and 2022 Baseline Values and Assumptions Documents (Table 2)^39,40^.

Cold storage (CS^ESM^), water (W^ESM^), and waste storage (WS^ESM^) used equivalency factors derived from non-crew time ESM values for baseline technologies described in nominal Mars surface missions^39,61^. These working mass equivalency factor calculations for Eq. 1 are detailed in Supplementary data 1. Briefly, the cold storage equivalency (CS_eq_) assumed a density of 1000 kg m^−3^ for mass requiring cold storage and a 0.614 m^3^ internal volume freezer analogous to the current International Space Station (ISS) system^39^. The Mars mission ESM of this freezer system was estimated at 490 kg eq. The freezer ESM was divided by the internal volume and multiplied by the density of the stored mass to obtain a CS_eq_ of 0.80 kg eq kg^−1^ for cold-stored mass.

The water equivalency (*W*_eq_) and waste storage equivalency (WS_eq_) factors were calculated from non-crew time ESM divided by the estimated total mission load using a Mars mission scenario where a crew of six used 30 kg of water per crew member per day and produced 1.5 kg solid waste per crew member per day^61^. The non-crew time W^ESM^ was calculated at 12,800 kg eq, assuming a water processor similar to the ISS water treatment system^61^. A non-crew time WS^ESM^ of 4480 kg eq was calculated from the mass, volume, power, and cooling estimates for a baseline solid waste disposal system proposed for a Mars surface habitat^61^. The equivalency factors of 0.12 kg eq kg^−1^ for *W*_eq_ and 0.83 kg eq kg^−1^ for WS_eq_ were derived from the calculated ESM of each system and divided by the estimates of total use during 600 days of surface operations. Detailed assumptions and ESM calculations for Figs. 2, 3 are given in Supplementary data 1, and detailed calculations for the three designs are given in Supplementary data 2.

### SCM metric

SCM was calculated by summing the number of components and the number of one-way interactions between components^22^. Components are integrated subsystems that perform one or more bioprocessing steps, such as equipment available from commercial vendors. Auxiliary parts such as valves, ordinary filters, and sensors were excluded with the rationale that integration of major components account for most of the system complexity, cost, and failure modes^22^. The number of one-way interactions is derived from a top-level bioprocessing system block diagram based on physical connections between components (Fig. 6).

### TRL and IRL metrics

TRL and IRL metrics were assigned for each of the dewatering, cell lysis, and purification options included in the three designs using existing guidelines^32–34^. TRL is a numerical scoring system for ranking the relative risk of advancing development of technologies from fundamental principles to flight proven hardware systems. TRL levels were assigned to each technology considered using the following scoring: TRL 1 basic principles, TRL 2 application concept formulated, TRL 3 proof of concept, TRL 4 laboratory breadboard validated, TRL 5 breadboard validated in relevant environment, TRL 6 prototype demonstrated in operational environment, TRL 7 system demonstrated in operational environment, TRL 8 system flight qualified, and TRL 9 system flight proven^32^.

IRL is a similar scoring metric designed to assess risks associated with integration of technologies from identifying interfaces to demonstrating integration in flight^50^. IRL levels were assigned to each technology considered the following scoring: IRL 1 interfaces identified, IRL 2 interaction characterized, IRL 3 communication compatibility shown, IRL 4 integration data quality checked, IRL 5 control established, IRL 6 information exchange established, IRL 7 integration requirements validated, IRL 8 mission qualified, and IRL 9 mission proven^33,34^.

### Degree of crew assistance metric

The degree of crew assistance was estimated based on whether the commercially available components were integrated and easily automatable. Crew support was assumed if commercial technology was not readily available to automate a specific step in the proposed workflow of each design. The sum of crew assisted steps was the degree of crew assistance for the design.

### Dewatering technologies

Tangential flow filtration, batch centrifugation, and counterflow centrifugation were three dewatering methods compared for this analysis. The dimensions and mass of the tangential flow filter were based on Xampler cartridge with 1 mm fiber diameter, 500 kDa pore size and 30 cm path length with a 3 M housing (Cytiva, Marlborough, MA, USA). To control fluid flow through the tangential flow filter, an Ismatec Reglo ICC Digital Pump with 4-Channels and 8-Rollers (Cole-Palmer, Vernon Hills, IL, USA) was included in the ESM calculations. A modified version of the Drucker model 755VES swinging bucket centrifuge (Drucker Diagnostics, Port Matilda, PA, USA) has been approved for operation on the ISS, and the specifications of the commercially available centrifuge were used to calculate the ESM. Twenty disposable 50 mL tubes were assumed to harvest the 1 L culture volume during every run. The counterflow centrifuge data was based on specifications for the CTS Rotea Counterflow Centrifugation System (Thermo Fisher Scientific, Waltham, MA, USA).

### Lysis technologies

The methods considered for lysis were bead beating, enzymatic lysis, a flow-through sonicator, and a large-volume probe sonicator. The bead beater analysis was based on the Claremont Biosolutions LLC (Upland, CA, USA) OmniLyse HL beadbeater flow-through lysis device. Although originally developed for small volumes, the OmniLyse HL unit was assumed to lyse large volumes with equal efficiency using extended processing times. The enzyme lysis protocol assumed that the biomass was incubated for 30 min at ambient temperature with 0.25 mg mL^−1^ lysozyme, 0.1 mL mL^−1^ Pierce universal nuclease (Thermo Fisher Scientific, Waltham, MA, USA), and 0.1% weight per volume Triton X at final concentration. Mass and volumes for ESM were calculated assuming the solid reagents had a density equal to NaCl (2.17 g cm^−3^), and liquid reagents had a density equal to water (1 g cm^−3^). The QSonica (Newton, CT, USA) ultrasound generator was modeled assuming a 1-inch replaceable tip for a large volume batch method and a Q500 FloCell unit for the flow through method.

### Protein purification technologies

Five His-tag affinity purification methods were compared using public domain product information available from commercial vendors. The Sigma Aldrich (St. Louis, MO, USA) His-Select Ni affinity gel was used to represent affinity columns requiring clarified lysates. The Cytiva (Marlborough, MA, USA) HisTrap FF was an exemplar of a crude lysate column that does not require clarification before sample loading. Batch resin purification specifications were estimated for the resin “teabag” method from Castaldo, et. al.^60^ Millipore Sigma (Burlington, MA, USA) HIS-Select® Nickel Magnetic Agarose Beads specifications were used to estimate the amount of resin required for batch purification with magnetic beads, while mass and volume of the beads were assumed to be equivalent to affinity resin. For affinity membrane purification, Capturem large volume filters (Takara Bio USA Inc., San Jose, CA, USA) were used to estimate the quantity of membrane required, while mass and volume of the membrane was assumed to be equivalent to Whatman filter paper (200 g cm^−2^).

The buffer composition was 20 mM HEPES, 500 mM NaCl and 20 mM imidazole for the binding buffer, and 20 mM HEPES, 500 mM NaCl and 200 mM imidazole for the elution buffer for all the methods. Buffer volumes were modeled using manufacturer protocols or the resin “teabag” method^60^.

### Bioprocessing system designs

All three post-growth bioprocessing system designs assumed a Claremont Biosolutions LLC (Upland, CA, USA) OmniLyse® HL disposable bead beater for in-line cell lysis from the biomass reservoir. The first design included a Thermo Fisher Scientific (Waltham, MA, USA) CTS Rotea Counterflow Centrifugation System with disposable, single use kits for processing. This counterflow centrifuge includes integrated pinch-valves, a peristaltic pump, and a controller for automation. QIAexpressionist Ni-NTA resin (Qiagen Inc., Valencia, CA, USA) was included for affinity purification.

The second design uses a MidGee ultrafiltration cartridge UFP-5-C-MM01A (Cytiva, Marlborough, MA, USA) and an Applikon Biotechnology (JG Delft, Netherlands) “my-control” with built in peristaltic pumps for biomass dewatering using tangential flow filtration and subsequent cell lysis. For protein purification, an Automate Scientific (Berkeley, CA, USA) Perfusion System and ValveLink8.2 Perfusion Controller were assumed to integrate with an Masterflex (Gelsenkirchen, Germany) L/S® Digital Drive peristaltic pump with an Easy-Load® 3 Pump Head for Precision Tubing. This fluid control system was assumed to automate affinity purification in a Cytiva 5 mL HisTrap FF crude lysate column (Marlborough, MA, USA).

Dewatering and lysis components in design 3 were identical to design 2. Protein purification was based on a non-commercial affinity belt system. Rollers were proposed to move an affinity membrane belt continuously through chambers containing the lysate, wash buffers, and elution buffers. The total volume of the system as assumed to be 0.005 m^3^ including the chambers, walls, and rollers. The mass was assumed to be 500 g. One Transmotec Inc. (Burlington, MA, USA) 12 V, 2 A DC motor was included to operate the rollers.

Disposable materials such as bags, and sterile filters for all three designs were estimated for the system based on commercial options and material properties, while the additional mass of tubing and luers were assumed to be 0.1 kg for all designs.

### Reporting summary

Further information on research design is available in the Nature Research Reporting Summary linked to this article.

### Supplementary information

Supplementary data 1

Supplementary data 2

Reporting Summary

## Data Availability

All source data and ESM calculations needed to replicate the study are provided in the manuscript and Supplementary data 1 and 2.
